# Cardiac Care of Non-COVID-19 Patients During the SARS-CoV-2 Pandemic: The Pivotal Role of CCTA

**DOI:** 10.3389/fcvm.2021.775115

**Published:** 2021-11-24

**Authors:** Edoardo Conte, Saima Mushtaq, Maria Elisabetta Mancini, Andrea Annoni, Alberto Formenti, Giuseppe Muscogiuri, Margherita Gaudenzi Asinelli, Carlo Gigante, Carlos Collet, Jeroen Sonck, Marco Guglielmo, Andrea Baggiano, Nicola Cosentino, Marialessia Denora, Marta Belmonte, Cecilia Agalbato, Andrea Alessandro Esposito, Emilio Assanelli, Antonio L. Bartorelli, Mauro Pepi, Gianluca Pontone, Daniele Andreini

**Affiliations:** ^1^Department of Biomedical Sciences for Health, University of Milan, Milan, Italy; ^2^Centro Cardiologico Monzino, Istituto di Ricerca e Cura a Carattere Scientifico, Milan, Italy; ^3^Cardiovascular Center Aalst, Onze-Lieve-Vrouwziekenhuis Hospital, Aalst, Belgium; ^4^Department of Advanced Biomedical Sciences, Federico II University of Naples, Naples, Italy; ^5^Foundation Istituto di Ricerca e Cura a Carattere Scientifico Ca' Granda, Ospedale Maggiore Policlinico, Milan, Italy; ^6^Department of Biomedical and Clinical Sciences “Luigi Sacco”, University of Milan, Milan, Italy; ^7^Department of Clinical Sciences and Community Health, Cardiovascular Section, University of Milan, Milan, Italy

**Keywords:** atherosclerosis, COVID-19, cardiac CT, chest pain, coronary artery disease

## Abstract

**Aim:** The aim of this study is to evaluate the potential use of coronary CT angiography (CCTA) as the sole available non-invasive diagnostic technique for suspected coronary artery disease (CAD) during the coronavirus disease 2019 (COVID-19) pandemic causing limited access to the hospital facilities.

**Methods and Results:** A consecutive cohort of patients with suspected stable CAD and clinical indication to non-invasive test was enrolled in a hub hospital in Milan, Italy, from March 9 to April 30, 2020. Outcome measures were obtained as follows: cardiac death, ST-elevation myocardial infarction (STEMI), non-ST-elevation myocardial infarction (NSTEMI), and unstable angina. All the changes in medical therapy following the result of CCTA were annotated. A total of 58 patients with a mean age of 64 ± 11 years (36 men and 22 women) were enrolled. CCTA showed no CAD in 14 patients (24.1%), non-obstructive CAD in 30 (51.7%) patients, and obstructive CAD in 14 (24.1%) patients. Invasive coronary angiography (ICA) was considered deferrable in 48 (82.8%) patients. No clinical events were recorded after a mean follow-up of 376.4 ± 32.1 days. Changes in the medical therapy were significantly more prevalent in patients with vs. those without CAD at CCTA.

**Conclusion:** The results of the study confirm the capability of CCTA to safely defer ICA in the majority of symptomatic patients and to correctly identify those with critical coronary stenoses necessitating coronary revascularization. This characteristic could be really helpful especially when the hospital resources are limited

## Introduction

The coronavirus disease 2019 (COVID-19) has rapidly and dramatically changed everyday life across the entire planet in an unprecedented way (1). In Italy, the first patient was presented at the end of February 2020 and was diagnosed nearby the metropolitan city of Milan in Lombardy, a region in the north of Italy (2). On March 7, 2020, almost all regions of northern Italy were locked down after the surge of the SARS-CoV-2 pandemic, and the public national healthcare system has been reorganized as a hub-and-spoke network (3). On Monday, March 9, 2020, the Centro Cardiologico Monzino, usually dedicated to cardiovascular care, was elected as a regional hub for cardiovascular emergencies, and all the non-urgent activities were suspended until April 30, 2020 (4).

Chest pain is a very common symptom that may subtend a wide range of clinical entities from non-cardiovascular and benign conditions to the acute coronary syndrome. Physical examination and rest ECG are the first steps in the clinical evaluation, but coronary artery disease (CAD) cannot be excluded in the patients with suspect symptoms by clinical assessment alone. Non-invasive diagnostic tests are recommended to establish the diagnosis and risk-stratify the patients (5). Before March 2020, the last version of ESC Guidelines on the management of chronic CAD recommended CCTA, stress cardiac magnetic resonance, and stress echocardiography at the same level of appropriateness (6–8). With the advent of the COVID-19 pandemic, cardiologists suddenly had to tackle a critical problem, namely, limited access to cardiovascular care and resources.

When compared to the pre-COVID era, during the first pandemic peak, in March 2020, non-invasive ischemic exercise/stress tests were not available in our center due to the extraordinary need to reorganize hospital activities and to the several concerns regarding the potential higher risk of contagion during exercise tests (due to hyperventilation and low-interpersonal distances without wide availability of the face mask and nasopharyngeal swab). Thus, CCTA was the sole test for patients with suspected CAD that remain available in a non-acute setting, even during the most severe first peak of the SARS-CoV-2 pandemic.

Thus, the aim of this manuscript is to describe the diagnostic and prognostic role that CCTA had in our hospital as the sole non-invasive diagnostic test for symptomatic patients with suspected stable CAD during an emergency pandemic when access to hospital facilities was limited.

## Materials and Methods

From March 9 to April 30th, during the peak of the COVID-19 pandemic, a consecutive cohort of the patients with high clinical suspicion of stable CAD and who underwent CCTA was enrolled in our cardiovascular dedicated hub hospital. It should be underlined that all the patients with highly suspected, but unknown, CAD evaluated at our center from March 9th and April 30 underwent CCTA as it was the only non-invasive test available for the suspected stable CAD in a non-acute setting, and invasive coronary angiography was almost entirely dedicated to the patients with the acute coronary syndrome. All the patients were evaluated for the presence of traditional cardiovascular risk factors, such as diabetes mellitus (glucose level of > 7 mmol/l, or the need for insulin, or oral hypoglycemic agents), hypercholesterolemia (total cholesterol level > 5 mmol/l or treatment with lipid-lowering drugs), hypertension (blood pressure > 140/90 mmHg or use of antihypertensive medications), positive family history of CAD [presence of CAD in the first-degree relatives younger than 55 years (male) or 65 years (female)], and currently smoking (5). All the patients provided written informed consent, and the local ethics committee approved the study.

Patients underwent CCTA with a new generation 256-slice CT scanner (Revolution CT, GE Healthcare, Milwaukee, WI, USA) that was performed according to updated international guidelines (9, 10) with the following parameters: slice configuration 256 × 0.625 mm, gantry rotation time 280 ms, and prospective ECG triggering. Tube current and tube voltage were adapted to BMI. Patients received a 50 ml (for BMI ≤ 25 kg/m^2^) or 60 ml (for BMI > 25 kg/m^2^) bolus of contrast medium (Iomeron 400 mg/ml, Bracco, Milan, Italy). All the patients received sublingual nitrates and betablockers (up to 25 mg of the intravenous metoprolol) before the CT scan.

Datasets of CCTA images were analyzed using vessel analysis software (CardioQ3 Package-GE Healthcare, Milwaukee, WI, USA). Reconstructed images were evaluated independently by two readers, both with over 10 years of clinical experience in the CCTA performance. Coronary arteries were divided into 16 segments according to the American Heart Association classification (11). In the case of motion artifacts with standard reconstruction, an additional reconstruction using an intracycle motion correction algorithm (a vendor-specific algorithm) was performed and analyzed. In case of image quality improvement after motion correction, the reconstructed image was used for analysis. Coronary segments were evaluated for the presence of critical stenoses, defined as coronary lumen narrowing exceeding 90%, and for the obstructive stenoses, defined as coronary lumen narrowing exceeding 50% (12). The presence of non-obstructive (from 0 to 50% stenosis) stenoses was recorded as well. For any disagreement in data analysis between the two readers, consensus agreement was achieved.

**Figure 1:**
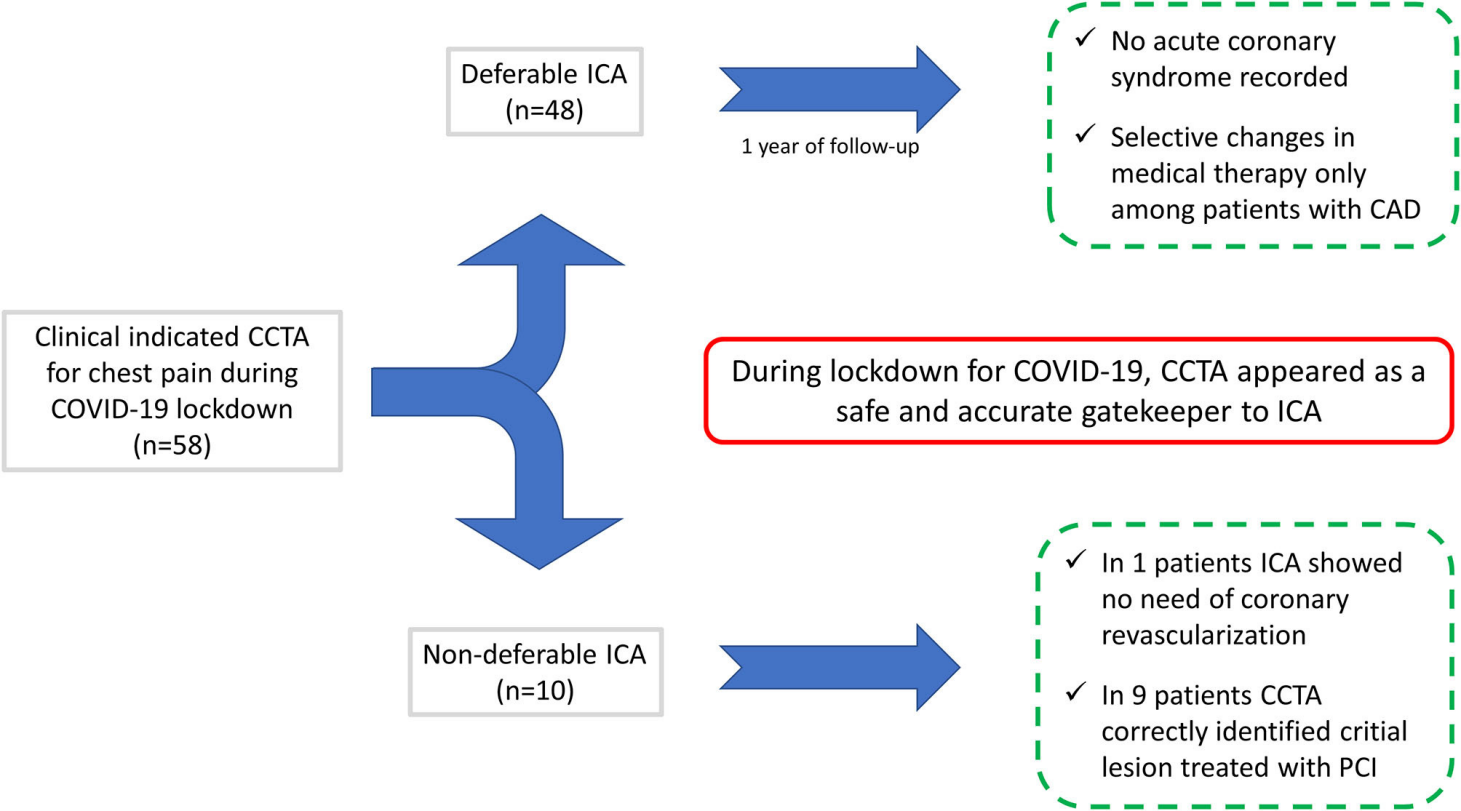
Coronary computed tomography angiography (CCTA) enabled the correct detection of the selected patients who needed non-deferrable treatment, while safely ruling out the critical coronary stenoses in 48 out of 58 patients who were free of the cardiovascular events at follow-up. CCTA, coronary computed tomography angiography; ICA, invasive coronary angiography; PCI, percutaneous coronary intervention.

When a clinical significant coronary stenosis (defined as >70% stenosis on a proximal coronary segment or >90% stenosis on any coronary segment) was detected at CCTA, the referring physician (cardiologist) was informed and, if the clinically indicated, an invasive coronary angiography (ICA) was scheduled (Figure 1). As routinely performed, myocardial revascularization for the coronary lesion <90% stenosis was performed only after invasive fraction flow reserve (FFR) resulted in being <0.8. Clinical follow-up was recorded by telephone interview, and medical records were screened for the patients in whom ICA was considered deferrable or not indicated after CCTA. Outcome measures were obtained as follows: cardiac death, ST-elevation myocardial infarction (STEMI), non-ST-elevation myocardial infarction (NSTEMI), and unstable angina defined according to ESC guidelines (13). All the changes in medical therapy following the result of CCTA were annotated.

The effective dose (ED) of CCTA was calculated according to the European Working Group for Guidelines on Quality Criteria in CT. The dose-length product (DLP) was measured in mGy × cm in each patient. The ED was calculated as the DLP times a conversion coefficient for the chest (*K* = 0.014 mSv/mGy × cm) (14).

## Statistical Analysis

Continuous variables were expressed as mean ± SD and discrete variables as absolute numbers and percentages. The Student's *t*-test was used to test differences in continuous variables between the two groups, and the chi-squared test or Fisher's exact test was used to assess differences regarding categorical data. Statistical significance was defined as a *p* < 0.05. Statistical analysis was performed using MedCalc Statistical Software version 19.2.1 (MedCalc Software Ltd, Ostend, Belgium; https://www.medcalc.org; 2020).

## Results

A total of 58 patients with a mean age of 64 ± 11 years (36 men and 22 women) were enrolled during the lockdown period for the COVID-19 pandemic. None of the patients suffered fever or respiratory symptoms suggestive of the SARS-CoV-2 infection. One patient showed bilateral ground-glass lung alterations on CCTA presumably due to the recent asymptomatic COVID-19 infection. Subsequent nasopharyngeal swab resulted negative for the SARS-CoV-2. Among the entire population enrolled, 10 (17.2%) patients underwent clinically indicated ICA according to CCTA findings, while ICA was considered deferrable in 48 (82.8%) patients. One patient was in atrial fibrillation during CCTA acquisition. A mean follow-up of 376.4 ± 32.1 days was obtained (Figure 1). No adverse events were recorded during or after CCTA. The mean radiation dose reached 4.7 mSv.

All the patients enrolled presented with symptoms highly suggestive for a new diagnosis of stable CAD, the mean pretest probability for CAD was 29.7% and resulted significantly higher among those who subsequently underwent ICA vs. those who did not (41.5 vs. 25.1%, respectively, *p* < 0.001) (Table 1). A total of 18 patients (31%) had typical chest pain that was significantly more prevalent among ICA vs. non-ICA group (80 vs. 20.8%, respectively, *p* < 0.001) (Table 1).

**Table 1:** Population characteristics.

	**Total population**	**Deferrable ICA**	**Non-deferrable ICA**	***p***
	**(*n* = 58)**	**(*n* = 48)**	**(*n* = 10)**	
**Clinical characteristics**				
Age, mean ± SD	64.7 ± 11.6	64.3 ± 11	66.3 ± 14.7	0.597
Sex, *n* (%)	36 (62)	28 (58.3)	8 (80)	0.207
BMI, mean ± SD	26.4 ± 4.5	26.6 ± 4.5	25.7 ± 5.2	0.524
Hypertension, *n* (%)	33 (56.8)	26 (54.2)	7 (70)	0.637
Dyslipidemia, *n* (%)	28 (48.2	23 (47.9)	5 (50)	0.904
Family history, *n* (%)	20 (34.4)	17 (35.4)	3 (30)	0.745
Diabetes, *n* (%)	7 (12)	4 (8.3)	3 (30)	0.058
Active smoking, *n* (%)	7 (12)	6 (12.5)	1 (10)	0.826
Past smoking, *n* (%)	22 (37.9)	19 (39.6)	3 (30)	0.573
Typical chest pain, *n* (%)	18 (31)	10 (20.8)	8 (80)	<0.001
Atypical chest pain, *n* (%)	32 (55.2)	29 (60.4)	3 (30)	0.081
Non-cardiac chest pain, *n* (%)	3 (5.2)	3 (6.2)	0	0.421
Dyspnea, *n* (%)	5 (8.6)	6 (12.5)	0	0.228
Pretest probability of CAD (%), mean ± SD	27.9 ± 14.3	25.1 ± 12.3	41.5 ± 16.2	<0.001
**CCTA**				
No CAD, *n* (%)	14 (24.1)	14 (29.2)	0	<0.001
Non-obstructive CAD, *n* (%)	30 (51.8)	29 (60.4)	1 (10)	<0.001
Obstructive CAD, *n* (%)	14 (24.1)	5 (10.4)	9 (90)	<0.001
Stenosis >90%, *n* (%)	6 (10.3)	0 (0)	6 (60)	<0.001
Radiation dose (mSV), mean ± SD	4.7 ± 2.2	4.7 ± 2.1	4.6 ± 2.6	0.786

*CCTA, coronary computed tomography angiography; CAD, coronary artery disease; ICA, invasive coronary angiography; BMI, body mass index*.

**Figure 2:**
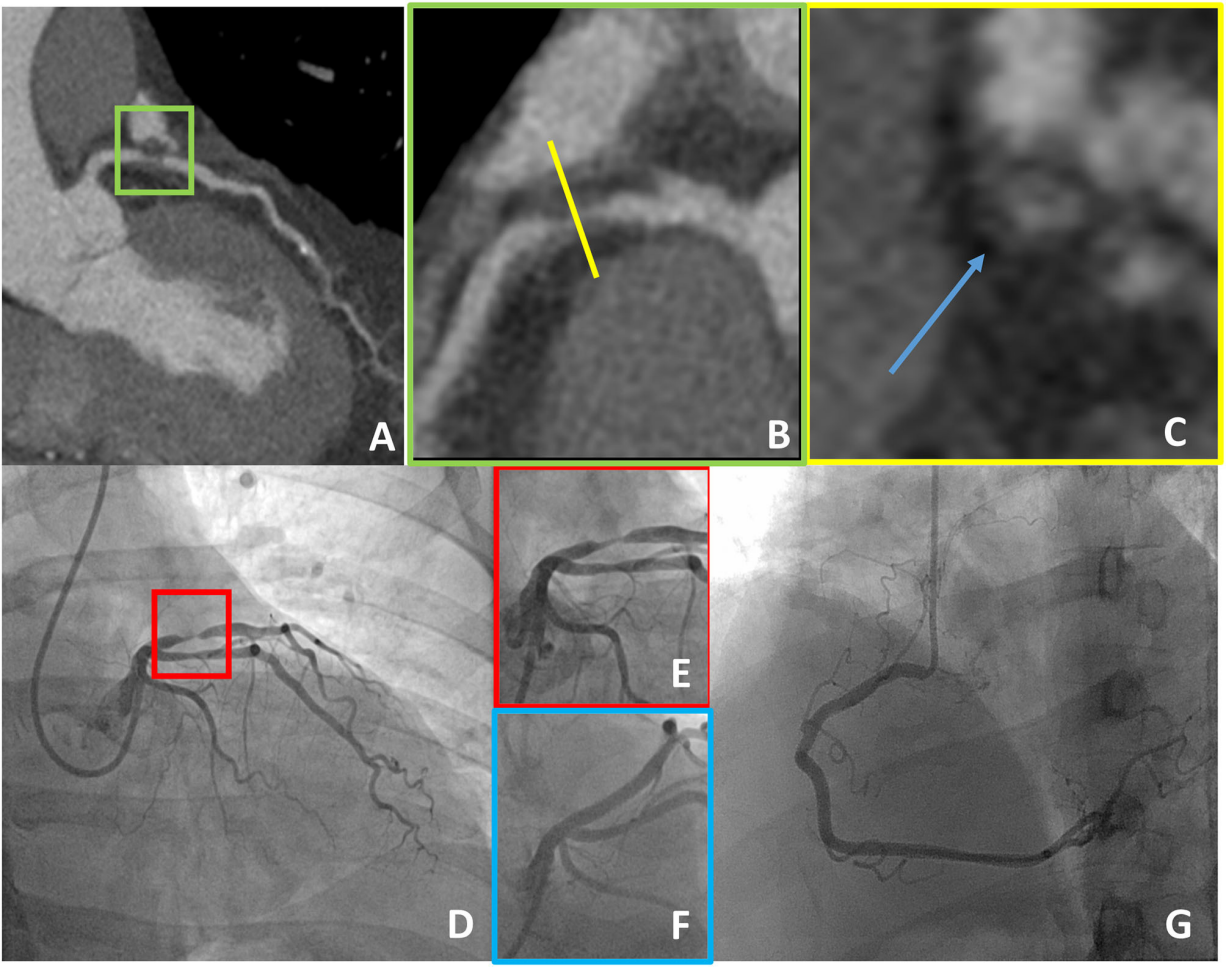
A case example of a 55-year-old man with the typical chest pain in whom CCTA detected critical stenosis of the proximal LAD **(A,B)**, showing at the same time high-risk plaque features (positive remodeling and low-attenuation plaque as demonstrated in a short axis view, blue arrow in **C**). The patient underwent ICA that confirmed subocclusive disease of the proximal LAD that was treated with PCI **(D–G)**. CCTA, coronary computed tomography angiography; LAD, left anterior descending artery; ICA, invasive coronary angiography; PCI, percutaneous coronary intervention.

Coronary CT angiography showed no CAD in 14 patients (24.1%), non-obstructive CAD in 30 (51.7%) patients, and obstructive CAD in 14 (24.1%) patients. None of the patients with normal coronary arteries at CCTA was sent to the catheterization laboratory for non-deferrable ICA (Table 1). A total of 10 patients were sent to ICA based on the CCTA results and in all but one severe CAD was confirmed and treated accordingly. CCTA showed critical/subocclusive (>90% diameter stenosis) lesions in six patients. All underwent percutaneous revascularization after ICA confirming the CCTA findings (Figure 2). The only patient who was not revascularized had a calcified non-high risk plaque of the proximal left anterior descending artery (LAD) and was referred to ICA due to typical angina with suspected left main CAD (Table 2).

**Table 2:** Clinical, CCTA, and ICA characteristics of patients who underwent non-deferrable ICA.

**Age and sex**	**Indication to CCTA**	**CCTA findings**	**High risk plaque features at CCTA**	**Clinical indication to ICA after CCTA results**	**ICA findings**	**Treatment**
79 y/o, male	Typical chest pain	40% stenosis of LM and 70% stenosis of proximal LAD	No	Symptomatic patient with at least moderate coronary stenosis and typical angina	40% stenosis of LM and 50% stenosis of proximal LAD	Medical therapy for stable CAD
47 y/o male	Atypical chest pain	70% stenosis of mid-LAD	PRI, LAP	Symptomatic patient with severe stenosis at CCTA	70% stenosis of mid-LAD	Percutaneous revascularization and drug-eluting stent implantation on mid-LAD
48 y/o male	Atypical chest pain	75% stenosis of mid-LAD. Moderate stenosis of LCX and RCA	PRI, LAP	Symptomatic patient with severe stenosis at CCTA	75% stenosis of mid-LAD	Percutaneous revascularization and drug-eluting stent implantation on mid-LAD
73 y/o, male	Typical chest pain	75% stenosis of mid-LAD	PRI, LAP	Symptomatic patient with severe stenosis at CCTA	75% stenosis of mid-LAD	Percutaneous revascularization and drug-eluting stent implantation on mid-LAD
55 y/o, male	Typical chest pain	99% stenosis of proximal LAD	PRI, LAP	Symptomatic patient with severe stenosis at CCTA	99% stenosis of proximal LAD	Percutaneous revascularization and drug-eluting stent implantation on proximal LAD
72 y/o, male	Typical chest pain	95% stenosis of diagonal branch	PRI, LAP	Symptomatic patient with severe stenosis at CCTA	90% stenosis of diagonal branch	Percutaneous revascularization and drug-eluting stent implantation on diagonal branch
86 y/o, female	Typical chest pain	99% stenosis of proximal LAD. Moderate stenosis of LCX	PRI, SC	Symptomatic patient with severe stenosis at CCTA	99% stenosis of proximal LAD. Moderate stenosis of LCX	Percutaneous revascularization and drug-eluting stent implantation on proximal LAD
55 y/o, male	Typical chest pain	99% stenosis of proximal LAD.	PRI, LAP, NRS	Symptomatic patient with severe stenosis at CCTA	99% stenosis of proximal LAD.	Percutaneous revascularization and drug-eluting stent implantation on proximal LAD
85 y/o, male	Typical chest pain	90% stenosis of mid-RCA	PRI, LAP	Symptomatic patient with severe stenosis at CCTA	90% stenosis of mid-RCA	Percutaneous revascularization and drug-eluting stent implantation on mid-RCA
63 y/o, female	Typical chest pain	75% stenosis of mid LAD	LAP	Symptomatic patient with severe stenosis at CCTA	75% stenosis of mid-LAD	Percutaneous revascularization and drug-eluting stent implantation on proximal LAD

*CCTA, Coronary computed tomography angiography; ICA, invasive coronary angiography; LAD, left anterior descendent artery; LCX, left circumflex; RCA, right coronary artery; PRI, positive remodeling index; LAP, low attenuation plaque; NRS, napkin ring sign*.

**Figure 3:**
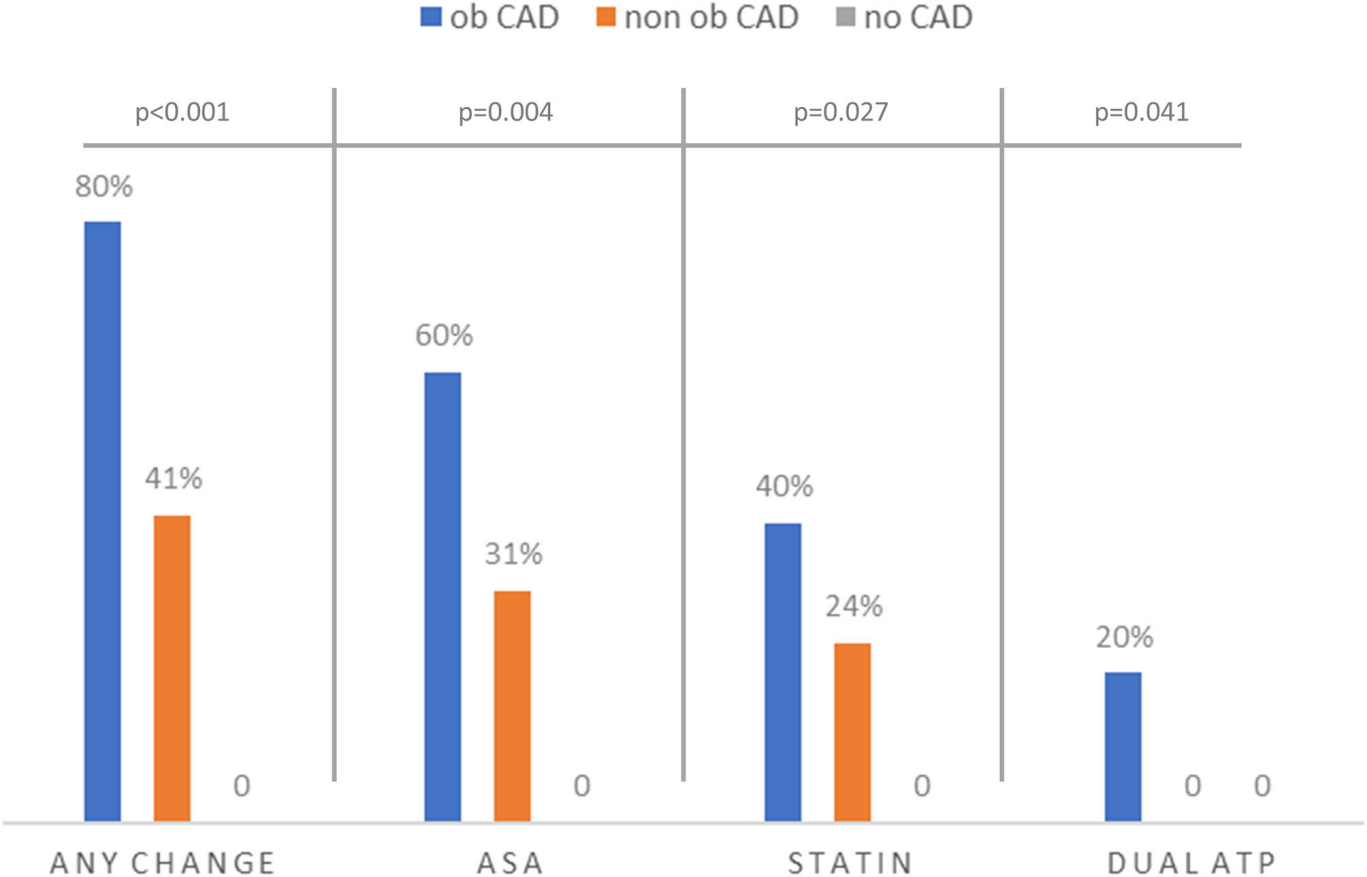
Prevalence of medical therapy changes according to CCTA findings among patients who did not undergo ICA. Of note, no new drugs were introduced by the referring physician in patients who were free of coronary atherosclerosis, while a significantly higher rate of aspirin and statin new prescriptions was observed when non-obstructive or obstructive CAD was identified at CCTA. Ob CAD, obstructive CAD; non-ob CAD, non-obstructive CAD; CAD, coronary artery disease; DAPT, dual antiplatelet therapy.

In 48 patients, there was no clinical indication for ICA. Fourteen patients (29.2%) showed normal coronary arteries at CCTA while non-obstructive (0–50% stenosis) and obstructive CAD (more than 50% stenosis) was demonstrated in 29 (60.4%) and five (10.4%) patients, respectively. Of note, no clinical events were recorded among the patients in whom ICA was considered not indicated or deferrable. Moreover, medical therapy was changed in 16 patients, which led to the symptomatic improvement in 13 patients (81.2%). No therapy change was recorded among the patients in whom CCTA excluded coronary atherosclerosis. Of note, medical therapy changes were significantly more prevalent in the patients with obstructive or non-obstructive CAD at CCTA. In 41% of the patients with non-obstructive CAD, medical therapy was modified, and more specifically, in nine (31%) and in seven (24%) of them, aspirin and statin therapy were prescribed (Figure 3).

## Discussion

To the best of our knowledge, this study is the first to describe the potential use of CCTA as the solely available gatekeeper for ICA in stable patients with chest pain with high clinical suspicion of CAD during the lockdown phase of the COVID-19 pandemic. Even if limited by the low number of patients enrolled, the results of the study appeared to confirm the capability of CCTA to safely defer ICA in the majority of the symptomatic patients and to correctly identify those with critical coronary stenoses necessitating coronary revascularization. This resulted to be of the utmost importance taking into consideration the need to limit hospital access to non-COVID patients. Moreover, the identification of non-critical atherosclerosis enabled physicians to optimize medical therapy in a well-selected subgroup of patients (15).

The advent of SARS-CoV-2 infection dramatically changed cardiovascular care and management with healthcare resources mostly focused on the patients with COVID-19 (16). During the lockdown phase, people were advised to avoid, if possible, emergency departments that were overwhelmed by patients with COVID-19. Moreover, there was a general reluctance to go to the hospital for the SARS-CoV-2 infection fear. Consequently, a reduced rate of hospital admission was observed with a potential increase of cardiac mortality from ACS due to the lower medical referrals (17). On the contrary, in this cohort, no cardiovascular were recorded at mid-term follow-up among patients in which ICA was deferred, while all but one patient with non-deferable ICA according to CCTA underwent appropriate myocardial revascularization.

Thus, the results of this study suggest that, due to its high-negative predictive value for obstructive CAD (8), CCTA correctly identified the great majority of the patients in whom ICA could be apparently safely deferred (82% of the patients) avoiding overcrowded hospitals and emergency departments, especially during a pandemic surge. However, it should be underlined that both the low number of patients enrolled and the absence of long-term follow-up represents a limitation to this study. Of interest, identification of non-obstructive CAD at CCTA has prognostic value (18–20) and, as previously demonstrated, should lead to optimal medical therapy implementation, further improving the prognosis of the patient (15). In our study, no invasive imaging was recommended to patients with normal coronaries at CCTA, avoiding unnecessary hospitalization in time of the limited resources. On the contrary, 41% of the patients with non-obstructive CAD had their medical therapy optimized.

On the other hand, CCTA permitted correctly identifying patients with severe coronary stenoses necessitating non-deferrable treatment (18% of the patients). Upon the detection of severe disease by CCTA, the referring physician (cardiologist) was immediately informed, and patients were managed in a dedicated non-COVID-19 pathway and catheterization laboratory, lowering the probability of SARS-CoV-2 infection while providing at the same time the best treatment strategy and reducing the risk of subsequent ACS (21).

A COVID-19 pandemic is a generation-defining event, and cardiovascular imaging practice has been deeply impacted as well (22–26). The results of this observational study suggest that CCTA is an appropriate and safe tool for the non-invasive evaluation of the suspected CAD when facing limited access to cardiovascular care and resources. Indeed, compared with the other non-invasive diagnostic tools, CCTA requires only a minimal time of contact between patients and healthcare professionals.

## Study Limitation

This study has several limitations. First, only the patients with a clinical indication underwent ICA, leading to the potentially underestimated false-negative results of CCTA. However, no clinical events were recorded during follow-up among the patients who did not undergo ICA. In this regard, it should be underlined that no further cardiac imaging was carried out in the follow-up period, thus, it was not possible to certainly exclude myocardial damage occurrence during follow-up; however, no major symptoms suspected for the cardiovascular events were recorded at follow-up.

Second, there the absence of another non-invasive control group, the low number of patients enrolled and the midterm follow-up may undermine the scientific strength of our findings, which should be considered as of speculative nature. Third, a larger cohort and a longer follow-up are needed for the validation of this report. Nevertheless, it should be considered that the present study has been performed during a global pandemic emergency with limited access to healthcare resources, and any control group randomly selected from the previous years could not be compared with the study population as the environmental conditions were totally different. Finally, we would like to highlight that the results of this study were obtained in a cardiovascular focused center using the last generation CT using postprocessing tools dedicated to the coronary analysis that may not be widely available, limiting the wide application study results in the different settings.

## Conclusion

We describe the potentially pivotal role of CCTA in the diagnostic pathway of patients with non-COVID-19 with chest pain due to suspected CAD during the SARS-CoV-2 pandemic. This non-invasive imaging tool enhanced the selection of patients for the ICA and potential revascularization during a lockdown period characterized by increased mortality due to delayed or deferred hospitalization of patients with CAD. The high-negative predictive value of CCTA enables to safely defer in-hospital care. Indeed, patients with non-obstructive CAD could be identified and safely treated by the referring physicians (cardiologists). On the contrary, CCTA helps in identifying patients who necessitate ICA ensuring adequate resource utilization during the pandemic.

## Data Availability Statement

The original contributions presented in the study are included in the article/supplementary materials, further inquiries can be directed to the corresponding author/s.

## Ethics Statement

The studies involving human participants were reviewed and approved by Ethics Committee of Centro Cardiologico Monzino, IRCCS. The patients/participants provided their written informed consent to participate in this study.

## Author Contributions

EC and DA conceptualized the manuscript. EC wrote the first draft of the manuscript. SM, MM, AA, AF, and GM performed and analyzed CT images. MGA, CG, MG, MD, MB, CA, and AB retrieved clinical data, follow-up information, and performed statistical analysis (data curation). CC, JS, and NC adjudicate events at follow-up. AE, EA, ALB, MP, GP, and DA provide senior expert advice and supervision. All authors contributed to the article and approved the submitted version.

## Conflict of Interest

The authors declare that the research was conducted in the absence of any commercial or financial relationships that could be construed as a potential conflict of interest.

## Publisher's Note

All claims expressed in this article are solely those of the authors and do not necessarily represent those of their affiliated organizations, or those of the publisher, the editors and the reviewers. Any product that may be evaluated in this article, or claim that may be made by its manufacturer, is not guaranteed or endorsed by the publisher.
